# Pediatric *Mycoplasma pneumoniae*-induced rash and mucositis in China: clinical spectrum, co-infections and risk factors for recurrence—a retrospective cohort study

**DOI:** 10.3389/falgy.2025.1646688

**Published:** 2025-10-01

**Authors:** Yang Wang, Lei Jiao, Lin Ma, Zigang Xu, Yuan Liang

**Affiliations:** Department of Dermatology, Beijing Children’s Hospital, Capital Medical University, National Center for Children’s Health Xicheng District, Beijing, China

**Keywords:** *Mycoplasma pneumoniae*–induced rash and mucositis, *Mycoplasma pneumoniae*, children, recurrent, co-infection, reactive infectious mucocutaneous eruption

## Abstract

**Background:**

*Mycoplasma pneumoniae*–induced rash and mucositis (MIRM) is a unique entity distinct from both erythema multiforme and Stevens-Johnson syndrome/toxic epidermal necrolysis. There are limited data on pediatric cases of MIRM in China.

**Objective:**

To evaluate the clinical characteristics and recurrence frequency of pediatric cases of MIRM and to summarize the co-infections beyond *M. pneumoniae* infection.

**Methods:**

This retrospective study was conducted through a chart review of patients with MIRM admitted to dermatology inpatient department from September 2017 to July 2021. Pediatric patients with MIRM 4 years to 12 years who met Canavan's criteria were included in the study.

**Results:**

A total of 23 patients with MIRM aged 7.86 ± 2.92 years were included. Oral mucosa was the most common site of mucosal involvement. Average number of involved mucous membranes was 2.83 ± 0.89. Average length of hospital stay was 10.30 ± 3.34 days. Length of hospital stay in recurrent cases was shorter than isolated cases (6.3 days vs. 10.17 days). Recurrence was observed in 21.7% of patients. The number of mucosal membranes involved was more in the first episode of recurrent cases than isolated cases (3.2 vs. 2.72). Of all patients, 47.8% were co-infected with pathogens apart from *M. pneumoniae*. Recurrence rate of the co-infection group was 36.4%.

**Conclusion:**

We report observations from the largest pediatric cohort with MIRM in China. Patients with younger age at onset had more severe skin and mucosal involvement, even similar to SJS/TEN. A higher recurrence rate and incidence of co-infections were observed in our cohort. The co-infection group had a higher recurrence rate, which further supports the concept of reactive infectious mucocutaneous eruption.

## Introduction

*Mycoplasma pneumoniae* (Mp), a common pathogen of respiratory infections in children, can induce extrapulmonary manifestations, such as pericarditis, thrombosis, hemolytic anemia, encephalitis, and mucocutaneous manifestations, in 25%–30% of cases (1, 2). The mucocutaneous eruptions associated with Mp was previously considered along the spectrum of erythema multiforme (EM), Stevens-Johnson syndrome (SJS) and toxic epidermal necrolysis (TEN). Mycoplasma pneumoniae-induced rash and mucositis (MIRM), a rare disease, is characterized by mucositis with eruptions that may be target-like and/or sparse vesiculobullous. The pathogenesis and the treatment pattern of MIRM are different from those of EM, SJS and TEN (3). In 2015, Canavan et al. proposed MIRM as a unique entity distinct from EM, SJS and TEN (4). The disease course of MIRM is milder and the rates of sequelae and mortality are lower as compared with SJS and TEN (2). Reports on mucocutaneous diseases induced by non-Mp pathogens alone or in conjunction with Mp have been published and has been termed reactive infectious mucocutaneous eruption (RIME) to broaden the spectrum of pathogens (5). Reactive infectious mucocutaneous eruption (RIME) is an umbrella term that includes all parainfectious, acute, mucosa-predominant eruptions (6). So far, only a few case series have been reported on MIRM in China (7).

We reviewed 23 pediatric cases of MIRM from Beijing Children's Hospital, focusing on the clinical characteristics and treatment in Chinese children, and compared the clinical differences between isolated cases with single episode and those with recurrent disease. In addition, we summarized the co-infections of these patients beyond Mp infection.

## Methods

### Patients

All patients from the dermatology inpatient consult service at Beijing Children's Hospital diagnosed with MIRM between September 2017 to July 2021 were reviewed. Pediatric patients aged 4–12 years were included in the study if they met the criteria proposed by Canavan (skin detachment <10% of body surface area; ≥2 mucosal sites involved; scattered atypical targets or few vesiculobullous lesions; and presence of Mp infection) (4). Patients were excluded if they failed to meet Canavan's criteria or if the overall clinical impression appeared to be more consistent with EM or SJS. A written informed consent was obtained from all pediatric patients (aged ≥7 years) and their parents. All data were deidentified.

### Study procedure

Patients were tested for Mp infection using IgM serology and nasopharyngeal PCR, and the infection was confirmed if the results of these tests were positive. Blood and respiratory secretions of all patients were tested for other infectious agents supposed to be involved (in favor of a recent infection or primary infection), and the presence of Epstein–Barr virus, cytomegalovirus, or human herpes virus in blood sample were tested using PCR. The follow-up period began at the time of the initial episode until the study end point in June 2024.

### Statistical analysis

Statistical analysis was performed using SPSS 27.0 software. Continuous variables are presented as the mean plus or minus SD and medians [interquartile ranges (IQR)] and the 3 group effects were determined by 1-way analysis of variance. Categorical variables are presented as frequencies (percentages). Specifically, non-parametric tests were chosen due to the small sample size and the potential for non-normally distributed data. The Kruskal–Wallis test was utilized for comparing three or more independent groups, while the Mann–Whitney *U* test was applied for two independent groups. A *P* < .05 was considered to be statistically significant.

## Results

**Figure 1:**
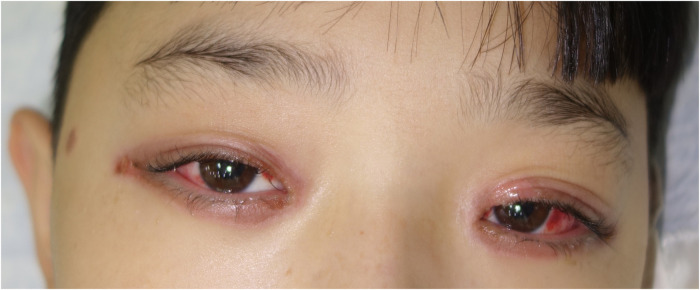
Ocular mucositis in a patient with *Mycoplasma pneumoniae*–induced rash and mucositis (MIRM).

**Figure 2:**
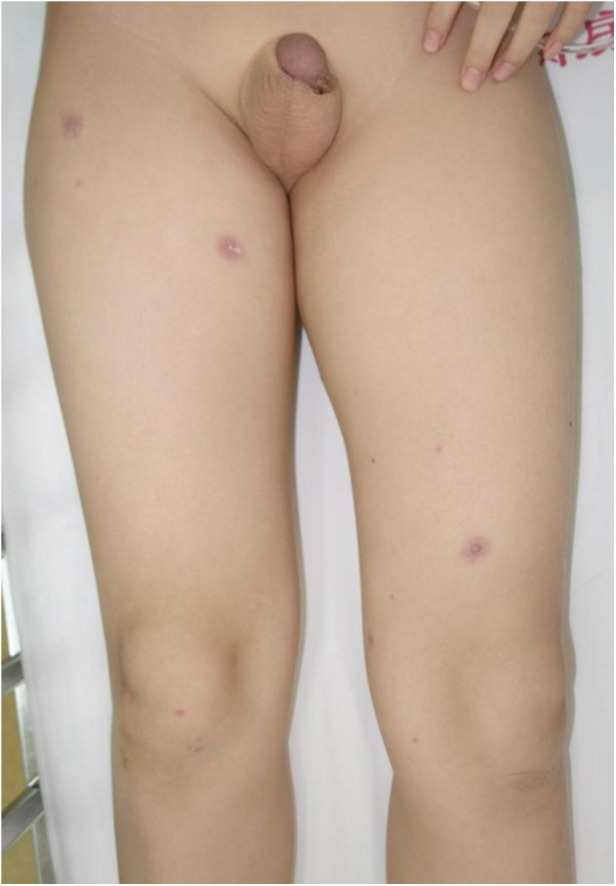
Oral mucositis in a patient with *Mycoplasma pneumoniae*–induced rash and mucositis (MIRM).

**Figure 3:**
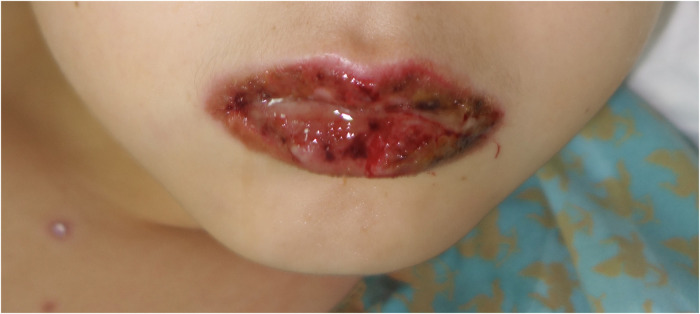
Skin lesions in the patient with *Mycoplasma pneumoniae*–induced rash and mucositis (MIRM).

A total of 23 patients (7 females and 16 males) were confirmed with diagnosis of MIRM. The manifestations of mucositis and skin lesions are shown in Figures 1–3. A summary of the clinical features of the patients with MIRM is presented in Online Repository Supplementary Table E1. The average ± standard deviation (SD) age of onset was 7.86 ± 2.92 years. Prodromal symptoms including fever (91.3%), cough (65.2%), sore throat (17.4%), and red eyes (4.3%) were observed in 20 (87.0%) patients. Sparse skin lesions were observed in 14 (60.9%) patients, and moderate skin lesions (with >10% of skin area being involved) were observed in 6 (26.1%) patients. Mucositis without rash was present only in 3 (13%) patients.

The average ± SD number of involved mucous membranes in these patients was 2.83 ± 0.89. The most common site of mucosal involvement was oral mucosa, occurring in every patient, followed by the ocular mucosa. At the initial onset, all patients were hospitalized and they received systemic antibiotics against Mp. Azithromycin was given to 22 (95.7%) patients and 1 (4.3%) patient received minocycline hydrochloride. β-Lactams (cefaclor and ceftriaxone) were given to 6 patients. Systemic corticosteroids were administered to 19 (82.6%) patients. These included the administration of either dexamethasone at a dose of 0.3–0.5 mg/kg/d or methylprednisolone/prednisone at a dose of 1–2 mg/kg/d. Intravenous immunoglobulins (IVIG) were administered to 18 (78.3%) patients at a dose of 400 mg/kg/d. Systemic corticosteroids and IVIG were used alone or in combination. The specific impact of different treatment options on prognosis was analyzed and no statistical significance was showed.

The average length of hospital stay was 10.30 ± 3.34 days. A total of 6 recurrences occurred in 5 (21.7%) patients, and the recurrence interval was 5 months to 1 year. Long sequelae including ophthalmic sequelae, bronchiolitis obliterans (BO) and oral adhesion were developed in 9 (39.1%) patients. Rare complications, such as myocarditis and pancreatic damage, occurred in 1 and 2 patients, respectively.

We compared the clinical characteristics of recurrent MIRM cases and single episode cases, as presented in Table 1. The age at initial onset in recurrent MIRM cases was older than that in isolated MIRM cases (9.06 years vs. 7.52 years). The first episode of recurrent MIRM cases had more involvement of mucosal membrane than isolated MIRM cases (number of mucosal sites involved: 3.2 vs. 2.72). The recurrent episodes of recurrent MIRM cases showed significantly fewer mucosal involvement (number of mucosal sites involved: 1.67) as compared with isolated MIRM cases (*P* = .012) and the first episode of recurrent MIRM cases (*P* = .005). The length of hospital stay for the recurrent episodes of recurrent MIRM cases was shorter (6.3 days) when compared with isolated MIRM cases (10.17 days). No significant differences were observed in length of hospital stay between the isolated MIRM cases (10.17 ± 2.79 days) and the first episode of recurrent MIRM cases (10.8 ± 5.26 days). However, the recurrent episodes of recurrent MIRM cases had markedly shorter length of hospital stay (6.33 ± 1.15 days) as compared with isolated MIRM cases (10.17 ± 2.79 days) and as compared with the first episode of recurrent MIRM cases (10.8 ± 5.26 days; *P* = 0.001). Although not statistically significant, the incidence of pneumonia and moderate skin lesions in single episode cases was higher as compared with recurrent cases. A positive chest image for diagnosing pneumonia was observed in 10 patients with a single episode and 1 patient with recurrent episodes.

**Table 1:** Clinical characteristics of recurrent and isolated MIRM cases.

Characteristic	Isolated MIRM^1^	First episode of recurrent MIRM^2^	Recurrent episodes of recurrent MIRM^3^	*P* value
	(*n* = 18)	(*n* = 5)	(*n* = 6)	
Age (yr), mean ± SD	7.52 ± 3.10	9.06 ± 1.89	9.52 ± 2.04	*P*1–2^a^ = .281
				*P*1–3^b^ = .138
				*P*2–3^c^ = .787
No. of mucosal sites involved, mean ± SD	2.72 ± 0.89	3.2 ± 0.84	1.67 ± 0.52	*P*1–2^a^ = .263
				*P*1–3^b^ = .012
				*P*2–3^c^ = .005
Length of hospital stay (days), mean ± SD	10.17 ± 2.79	10.8 ± 5.26	6.33 ± 1.15	*P*1–2^a^ = .781
				*P*1–3^b^ < .01
				*P*2–3^c^ = .001
Sex, *n* (%)				*P* = .554
Male	13 (72.2)	3 (60.0)	3 (50.0)	
Female	5 (27.8)	2 (40.0)	3 (50.0)	
Ethnic group, *n* (%)				*P* = .790
Han	16 (88.9)	4 (80.0)	5 (83.3)	
Mongolian	2 (11.1)	1 (20.0)	1 (16.7)	
Season, *n* (%)				*P* = .991
Spring	4 (22.2)	1 (20.0)	1 (16.7)	
Summer	3 (16.7)	1 (20.0)	2 (33.3)	
Autumn	4 (22.2)	1 (20.0)	1 (16.7)	
Winter	7 (38.9)	2 (40.0)	2 (33.3)	
Microbiological tests of co-infection beyond Mp, *n* (%)				*P* = .225
Yes	7 (38.9)	4 (80)	4 (66.7)	
No	11 (61.1)	1 (20)	2 (33.3)	
Skin detachment, *n* (%)				*P* = .269
Absent	2 (11.1)	1 (20)	2 (33.3)	
BSA < 10%	10 (55.6)	4 (80)	4 (66.7)	
10% ≤ BSA < 30%	6 (33.3)	0 (0)	0 (0)	
Positive chest image, *n* (%)				*P* = .200
Yes	10 (55.6)	1 (20.0)	1(16.7)	
No	8(44.4)	4(80.0)	5(83.3)	

*BSA*, body surface area; *MIRM*, *Mycoplasma pneumoniae*-induced rash and mucositis; *Mp, Mycoplasma pneumoniae*; *SD*, standard deviation.

a *P*1–2, *P* value between isolated MIRM cases and first episode of recurrent MIRM cases.

b *P*1–3, *P* value between isolated MIRM cases and recurrent episodes of recurrent MIRM cases.

c *P*2–3, *P* value between first episode and recurrent episodes of recurrent MIRM cases.

All patients tested positive for Mp IgM test at the initial and recurrent episodes; of them, 10 patients were positive for both Mp IgM and PCR tests at the initial episode. Moreover, positive antibodies against other pathogens such as group A streptococcus (GAS), influenza virus B and Chlamydia pneumonia (CP) were detected in 11 patients. Among them, 4 were recurrent cases and 7 were single episode cases. The incidence of co-infections were lower in single episode cases than in recurrent cases (8.3% vs. 36.4%) but was not statistically significant. Among these 4 recurrent cases, 3 had co-infections during their initial onset, while only one case had a co-infection at the time of recurrence. A summary of the types of co-infected pathogens in MIRM patients is presented in Online Repository Supplementary Table E2.

We compared the clinical characteristics of the group of patients who were infected with Mp alone with the group of patients with co-infection, as presented in Table 2. A significant difference was observed in the season of onset between the 2 groups (*P* = .037). In the co-infection group, 10 (90.9%) patients developed symptoms in autumn and winter. On the other hand, 8 (66.6%) patients who were infected with Mp alone developed symptoms in spring and summer. No significant statistical differences were observed in age of onset, length of hospital stays, and degree of skin and mucosal involvement between the 2 groups. The initial age of onset in the co-infection group was older than in the group infected with Mp alone (8.34 years vs. 7.42 years). The length of hospital stay in the group infected with Mp alone was longer than that in the co-infection group (11.25 days vs. 9.27 days). No significant difference was observed in the number of mucosal sites involved (2.75 vs. 2.90) between these 2 groups. Although the recurrence rate of the co-infection group was much higher than that of the Mp alone group, the difference between the 2 was not statistically significant (*P* = .155).

**Table 2:** Clinical characteristics of the group infected with Mp alone and the co-infected group.

Characteristic	The group infected with Mp alone (*n* = 12)	Co-infected group (*n* = 11)	*P* value
Age (yr), mean ± SD	7.42 ± 2.87	8.34 ± 3.03	.463
No. of mucosal sites involved, mean ± SD	2.75 ± 0.87	2.90 ± 0.94	.678
Length of hospital stay (days), mean ± SD	11.25 ± 2.37	9.27 ± 4.00	.160
Sex, *n* (%)			.193
Male	10 (83.3)	6 (54.5)	
Female	2 (16.6)	5 (45.5)	
Season, *n* (%)			.037
Spring	4 (33.3)	1 (9.1)	
Summer	4 (33.3)	0 (0)	
Autumn	2 (16.7)	3 (27.3)	
Winter	2 (16.7)	7 (63.6)	
Skin detachment, *n* (%)			.844
Absent	1 (8.3)	2 (18.2)	
BSA < 10%	8 (66.7)	6 (54.5)	
10% ≤ BSA < 30%	3 (25)	3 (27.3)	
Positive chest image, *n* (%)			.684
Yes	5 (41.7)	6 (54.5)	
No	7 (58.3)	5 (45.5)	
Full recovery, (*n*, %)			.684
Yes	5 (41.7)	6 (54.5)	
No	7 (58.3)	5 (45.5)	
Mucosal complications, (*n*, %)			.400
Yes	6 (50)	3 (27.3)	
No	6 (50)	8 (72.7)	
Recurrence, (*n*, %)			
Yes	1 (8.3)	4 (36.4)	.155
No	11(91.7)	7(63.6)	

*BSA*, body surface area; *Mp*, *Mycoplasma pneumoniae*; *SD*, standard deviation.

## Discussion

MIRM is a new concept describing mild skin lesions and severe mucosal symptoms that are induced by Mp infection (4). MIRM commonly affects children and young adolescents, and the frequency in children is about 6.8% (8).

To our knowledge, this study reports the largest pediatric series of MIRM in China. First, MIRM occurs more often in summer and early fall with male predominance, and the mean age of onset of MIRM is 11.96 ± 8.8years (4). Our study demonstrated that although the seasonal variability and male preponderance are comparable to prior reports (4), the mean age of onset in the present study cohort was younger (7.86 ± 2.92 years). In our cases, the proportion of skin detachment area exceeding 10% was higher than (26.1% vs. 19%), indicating that the skin involvement in MIRM is not always mild in pediatric patients (4), and may even be similar to SJS/TEN. The prodromal period of the cases reviewed in this study was shorter than that reported by Canavan et al. (4.33 ± 3.40 days vs. 8 ± 5 days) (4). The distribution of skin lesions was mainly generalized, followed by acral, which is different from that reported by Canavan et al. (4).

Second, in the present study cohort, the recurrence rate was 21.7%, which was different from the previous reports. Canavan et al. (4) reported a 8% recurrence rate, Liu et al. (7) reported a 20% recurrence rate and Liakos et al. (9) reported a 38% recurrence rate. Mixed recurrence rates could be related to the short-lived natural immunity of human body to Mp infection. In addition, according to the literature report, the recurrent MIRM has an increased frequency of human leukocyte antigens HLA-B51 and HLA-B27 (10), which supports the view that genetic factors might lead to skin and mucosal inflammatory responses to pathogens. Besides, the co-infected group had higher recurrence rate than the group infected with Mp alone (36.4% vs. 8.3%) in our study. Therefore, we speculated that co-infection of the MIRM in the initial onset may be a risk factor for recurrence. Previous report have shown that many patients with recurrence might have ≥2 relapses (9). However, we observed only one recurrence in most of our recurrent MIRM cases, and recurrence was reported twice in only one patient. The symptoms of recurrent MIRM cases at the recurrent episodes are milder than the first episodes both in the present study cohort and in previous studies (7, 9). This may be due to the possibility that pre-existing antibodies are present in the recurrent MIRM cases after the first episode, resulting in milder symptoms during the recurrence period. Notably, the patient who relapsed twice finally developed BO, which is a rare and severe sequela. This shows that although the symptoms of patients in recurrent episodes are milder than the first episodes, repeated mucosal damage may still lead to serious sequelae.

Third, for the first time, we report the incidence of co-infections with non-Mp pathogens in patients with MIRM. In this study cohort, 11 (47.8%) patients were co-infected with other pathogens apart from Mp. The coexistence of Mp with other agents of community-acquired bacterial pneumonia is very common, and such co-infections are found predominantly in children (1). The co-infection of Mp with other pathogens is more likely to occur in autumn and winter in our cases, which is different from Mp infections being more common during summer or early fall (1). In our study, the most common pathogens identified in co-infections were GAS and influenza virus B, both of which are prevalent during winter and spring. This result suggests that the onset of MIRM may influenced by other pathogens besides Mp. It was previously observed that mixed infections with bacteria and viruses tend to be associated with more severe illnesses (11). However, in our case, the there was no significant difference in the rash area and number of mucosal involvement between the co infected group and the group infected with Mp alone. Interestingly, we found that the recurrent MIRM cases had a higher incidence of co-infections with non-Mp pathogens than the isolated MIRM cases (80% vs. 38.9%). And the incidence of co-infections with non-Mp pathogens remains high in the recurrent episodes. Moreover, the recurrence rate of the co-infection group in our study cohort was much higher than the group infected with Mp alone (36.4% vs. 8.3%). Therefore, we reasonably speculate that co-infection with non-Mp pathogens plays an important role in both the first onset and the recurrence of MIRM.

In recent years, non-Mp pathogens have been reported to be implicated in the clinical presentation of post-infectious rash and mucositis (12–17). The umbrella term “RIME” was proposed to include non-Mp pathogens that may cause similar rashes with mucositis (5). Firstly, we hypothesize that co-infections may exacerbate the immune response by promoting increased immune complex deposition, which resulted in a higher recurrence rate. When multiple pathogens are present, the immune system generates a broader range of antibodies, potentially leading to the formation of more immune complexes. These immune complexes can deposit in mucosal tissues, activating complement and triggering an inflammatory cascade. Secondly, Co-infections can dysregulate cytokine production, leading to an exaggerated inflammatory response. Different pathogens may stimulate distinct cytokine profiles, which, when combined, could result in a more severe inflammatory milieu. Thirdly, Co-infecting pathogens may interact directly, either synergistically enhancing virulence or competing for resources. Such interactions could alter the clinical presentation and severity of MIRM. Besides, Co-infections may modify the host-pathogen interface, affecting pathogen adherence, invasion, and evasion of host defenses. This could lead to more extensive mucosal damage and prolonged disease course. Lastly, host genetic factors may influence susceptibility to co-infections and the severity of MIRM.

So far, there are no evidence-based guidelines on the treatment of MIRM. Given the similar clinical features, the treatment recommended for recurrent MIRM was same as the SJS. And the use of cyclosporine and TNF-α inhibitors have been successful in treating MIRM (18). Our patients received systemic treatments such as antibiotics (especially against Mp), corticosteroids, and IVIG in addition to supportive treatment. We had analyze the specific impact of treatment options on prognosis and found no statistical significance. We considered this may be related to the small sample size of this study, and further expansion of the sample size should be conducted in the future to analyze the impact of different treatment regimens on prognosis. Methods to prevent the recurrence of MIRM should be explored, in the view that most patients still have severe mucositis and require hospitalization when the disease relapses. In our study, many patients were co-infected with non-Mp pathogens, and the recurrence rate was much higher in the co-infection group. Therefore, we should also pay attention to other suspected pathogens besides Mp. Owing to the severe mucosal symptoms of MIRM, supportive treatment and multi-disciplinary consultations should be established.

## Conclusion

Previous reports suggest that MIRM is essentially an infection-reactive mucocutaneous disease that usually occurs in young children and has a favorable prognosis. However, we found that severe cases are not uncommon in pediatric cases. And we should also be alert to the occurrence of serious sequelae such as BO, especially in recurrent cases. We also found that patients with MIRM often have co-infections with pathogens other than Mp, which further supports the concept of RIME. And we found that the recurrence of MIRM may be related to co-infections beyond Mp infection. Additionally, we propose that co-infections may increase the risk of recurrence through immune complex deposition and complement activation, but acknowledge the lack of direct evidence. Clinicians should distinguish MIRM and SJS/TEN based on their etiology, but they cannot consider all MIRM as a disease with a good prognosis, especially for the recurrent MIRM patients. The limitation of our study is that it is a retrospective study with a small sample size, and there may be selection bias in single center data and a lack of control groups.

## Data Availability

The datasets presented in this article are not readily available because due to the fact that the raw data contains personal privacy, we regretfully cannot furnish the raw data. Nevertheless, we have meticulously presented a comprehensive account of the study design, analysis, results, procedures employed for data analysis and processing. Should the esteemed editor and reviewers require further elucidation or specific inquiries pertaining to the data, we pledge our utmost" commitment to providing detailed explanations and clarifications. Requests to access the datasets should be directed to Yang Wang, yangwang910521@163.com.
