# Supplementary material for: Pediatric Mycoplasma pneumoniae-induced rash and mucositis in China: clinical spectrum, co-infections and risk factors for recurrence—a retrospective cohort study
Source: Front Allergy. 2025 Oct 1;6:1646688. doi: 10.3389/falgy.2025.1646688 (PMC12521175; doi:10.3389/falgy.2025.1646688)
Supplement: Supplementary file 1 [file Table1.docx]

**Online repository Table E1.** The clinical features of patients with MIRM in this study cohort

|  | Total cohort (n=23) |
| --- | --- |
| **Age (yr), mean ± SD** | 7.86±2.92 |
| **Sex, n (%)** |  |
| Male | 16(69.6) |
| Female | 7(30.4) |
| **Ethnic group, n (%)** |  |
| Han | 20(87.0) |
| Mongolian | 3(13.0) |
| **Season, n (%)** |  |
| Spring | 5(21.7) |
| Summer | 4(17.4) |
| Autumn | 5(21.7) |
| Winter | 9(39.1) |
| **Positive Chest image, n (%)** | 11(47.8) |
| **Microbiological tests of co-infection beyond Mp, n (%)** | 11(47.8) |
| **Skin detachment, n (%)** |  |
| Absent | 3(13.0) |
| BSA<10% | 14(60.9) |
| 10% ≤ BSA < 30% | 6(26.1) |
| **Skin lesion distribution, n (%)** |  |
| Centripetal | 4(17.4) |
| Acral | 5(21.7) |
| Generalized | 11(47.8) |
| Absent | 3(13.0) |
| **No. of mucosal sites involved, mean ± SD** | 2.83±0.89 |
| **Mucous involvement site, n (%)** |  |
| Oral | 23(100) |
| Ocular | 22(95.7) |
| Urogenital | 15(65.2) |
| Anal | 9(39.1) |
| **Length of hospital stay (days), mean ± SD** | 10.30±3.34 |
| **Antibiotic, n (%)** |  |
| Azithromycin | 22(95.7) |
| Minocycline hydrochloride | 1(4.3) |
| Other antibiotics ^a^ | 6(26.1) |
| **Systemic corticosteroids^b^, n (%)** | 19(82.6) |
| **IVIG^c^, n (%)** | 18(78.3) |
| **Clinical outcomes, n (%)** |  |
| Full recovery | 11(47.8) |
| Mucosal complications ^d^ | 9(39.1) |
| Recurrence | 5 (21.7) |

*BSA,* Body surface area; *IVIG*, Intravenous immunoglobulins; *MIRM*, *Mycoplasma pneumoniae*-induced rash and mucositis; *Mp,* *Mycoplasma pneumoniae; SD*, Standard deviation.

^a^ 6 patients received β-Lactams (Cefaclor and ceftriaxone) for pulmonary symptoms.

^b^ Systemic corticosteroid was given at a dose of 0.3-0.5mg/kg/day (dexamethasone) or 1-2mg/kg/day (methylprednisolone or prednisone) for 7-14 days, then gradually reduced and withdrawn in 7-14 days.

^c^ IVIG was given at a dose of 400mg/kg/day for 3-5 days.

^d^ One patient developed BO and received low-dose azithromycin and prednisolone oral treatment for 6 months. One patient developed oral adhesions and was surgically treated. 9 patients got symptoms (xerophthalmia in 5 patients, conjunctivitis in 6 patients, keratitis in 1 patient, meibomian gland dysfunction in 1 patient).
