# Supplementary material for: Pediatric Mycoplasma pneumoniae-induced rash and mucositis in China: clinical spectrum, co-infections and risk factors for recurrence—a retrospective cohort study
Source: Front Allergy. 2025 Oct 1;6:1646688. doi: 10.3389/falgy.2025.1646688 (PMC12521175; doi:10.3389/falgy.2025.1646688)
Supplement: Supplementary file 2 [file Table2.docx]

**Online repository Table E2.** Types of co-infected pathogens in MIRM patients

| Case Number | First-episode | Recurrent episode |
| --- | --- | --- |
| Case 4 | GAS | Negative |
| Case 5 | GAS | NA |
| Case 6 | Influ. B | NA |
| Case 10 | Negative | Influ. B |
| Case 11 | GAS | Negative |
| Case 15 | GAS | NA |
| Case 16 | Influ. B | CP |
| Case 19 | Influ. B & GAS | NA |
| Case 20 | Influ. B & CP | NA |
| Case 21 | Influ. B & GAS | NA |
| Case 23 | Influ. B | NA |

GAS, group A streptococcus; Influ.B, influenza virus B; CP, Chlamydia pneumonia; *MIRM*, *Mycoplasma pneumoniae*-induced rash and mucositis; NA, not applicable; Cases 4, 10, 11, and 16 are recurrent cases.
